# Supplementary material for: Continental-scale suppression of an invasive pest by a host-specific parasitoid underlines both environmental and economic benefits of arthropod biological control
Source: PeerJ. 2018 Oct 19;6:e5796. doi: 10.7717/peerj.5796 (PMC6197050; doi:10.7717/peerj.5796)
Supplement: Supplemental Information 2 [file peerj-06-5796-s002.docx]

**Appendix A**

**Figure legends**

**Figure S1.** Region-specific patterns in field-level *P. manihoti* pest pressure (expressed as total number of mealybugs per 50 plants; Log10-transformed) and *A. lopezi* parasitism, as recorded at 110 different sites during the 2014-2017 dry season. Local parasitism rates are contrasted with established thresholds for successful biological control (Hawkins & Cornell 1994) (i.e., maxima of 33-36% for parasitoids in exotic locations), as depicted by the red box.

**Figure S2.** Temporal fluctuations in domestic (Thai Baht/kg) and export price (US$ FOB Bangkok) of cassava starch over a 2008-2016 period, covering the late 2008 *P. manihoti* invasion, the Nov. 2009 introduction of *A. lopezi* and subsequent region-wide distribution of the parasitoid (over 2010-2014). Data sourced from Thai Tapioca Starch Association *(http://www.thaitapiocastarch.org*).


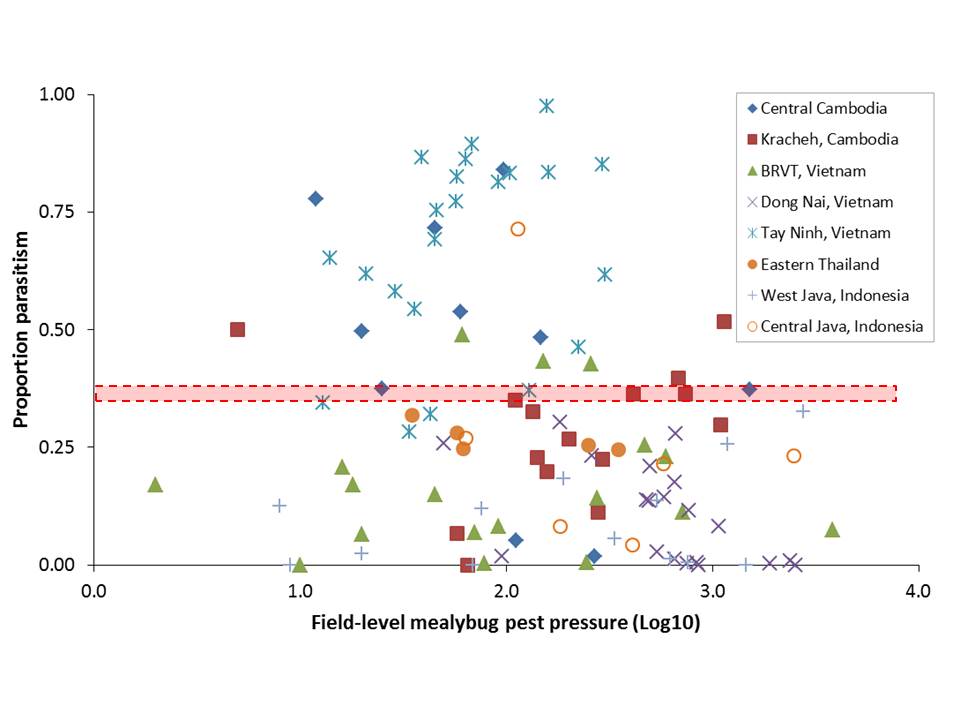


**Figure S1.**


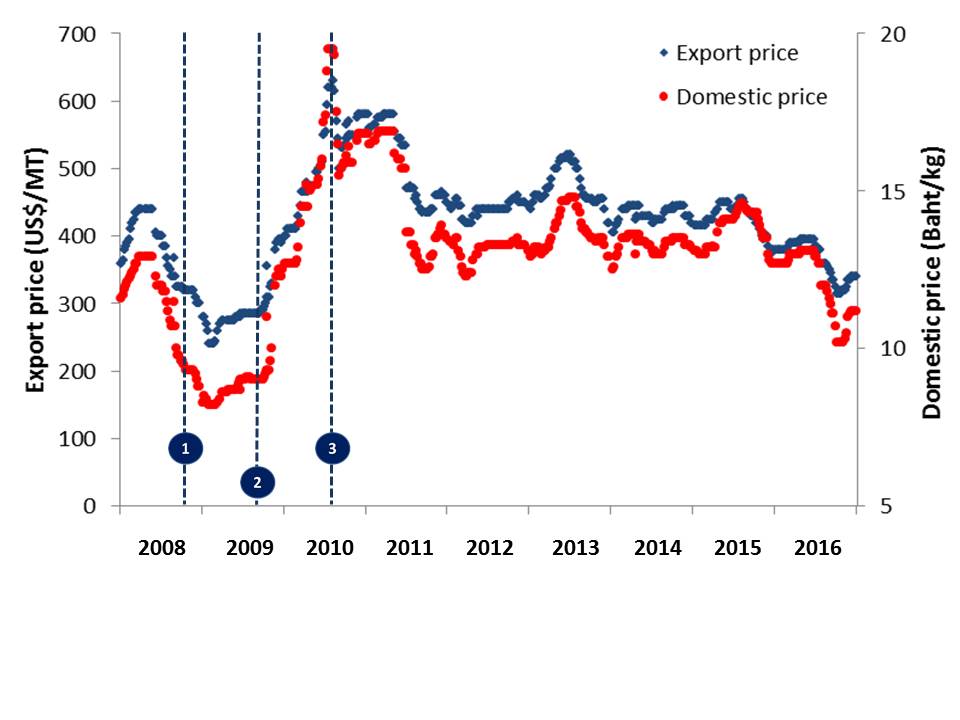


**Figure S2.**

**Table S1.** Additional information on model structure, describing all the models tested, and the output of the selected model (section *iv*)

| **Model** | **DF** | **AIC** | **Adjusted R2 values** | **p-value** | **Comments** |
| --- | --- | --- | --- | --- | --- |
| yield ~ time + rain + tmin + Int | 6 | 743.5 | 0.1 | 8.45E-05 | Model with the least AIC value obtained from step wise regression approach |
| **yield ~ time*Int + time*rain + time*tmin** | 9 | 719.3 | 0.22 | 3.78E-09 | Selected model |
| yield ~ time*Int + rain + tmin | 7 | 717 | 0.22 | 6.48E-10 |  |
| yield ~ Int + time*rain + tmin, data | 7 | 741.5 | 0.11 | 4.27E-05 |  |
| yield ~ Int + rain + time*tmin | 7 | 745.5 | 0.09 | 0.00024 |  |

**Yield**: yields measured across all regions

**Rain**: noted precipitation values across regions wherein yields were measured

**Tmin**: noted minimum temperature values across regions wherein yields were measured

**Int**: Corresponds to year of introduction of *Anagyrus* lopezi. “Absence” (dummy coded as 0) during the 2008, 2009 and 2010 growing seasons, and “Presence” (dummy coded as 1) for 2011 and 2012 growing seasons

**Time** : Corresponds to year for which the agro-climatic variables and yields were measured (i.e. from 2008 to 2012)

**DF**: Degrees of freedom

**AIC**: Akaike information criterion score
